# Static landscape features predict uplift locations for soaring birds across Europe

**DOI:** 10.1098/rsos.181440

**Published:** 2019-01-16

**Authors:** Martina Scacco, Andrea Flack, Olivier Duriez, Martin Wikelski, Kamran Safi

**Affiliations:** 1 Department of Migration and Immuno-ecology, Max Planck Institute for Ornithology, Am Obstberg 1, 78315 Radolfzell, Germany; 2 Centre d'Ecologie Fonctionnelle et Evolutive, UMR 5175 CNRS-Université de Montpellier- EPHE-Université Paul Valery, 1919 Route de Mende, 34293 Montpellier cedex 5, France; 3 Department of Biology, University of Konstanz, Universitätsstr. 10, 78464 Konstanz, Germany

**Keywords:** habitat suitability, movement ecology, random forest, species distribution model, anthropogenic infrastructure, energy landscape

## Abstract

Soaring flight is a remarkable adaptation to reduce movement costs by taking advantage of atmospheric uplifts. The movement pattern of soaring birds is shaped by the spatial and temporal availability and intensity of uplifts, which result from an interaction of local weather conditions with the underlying landscape structure. We used soaring flight locations and vertical speeds of an obligate soaring species, the white stork (*Ciconia ciconia*), as proxies for uplift availability and intensity. We then tested if static landscape features such as topography and land cover, instead of the commonly used weather information, could predict and map the occurrence and intensity of uplifts across Europe. We found that storks encountering fewer uplifts along their routes, as determined by static landscape features, suffered higher energy expenditures, approximated by their overall body dynamic acceleration. This result validates the use of static features as uplift predictors and suggests the existence of a direct link between energy expenditure and static landscape structure, thus far largely unquantified for flying animals. Our uplift availability map represents a computationally efficient proxy of the distribution of movement costs for soaring birds across the world's landscapes. It thus provides a base to explore the effects of changes in the landscape structure on the energy expenditure of soaring birds, identify low-cost movement corridors and ultimately inform the planning of anthropogenic developments.

## 1. Introduction

All animals interact with the surrounding environment, but for some of them the role of this environment becomes particularly relevant in constraining or supporting their movement. This especially applies to aerial or aquatic animals, whose movements actively modify and are, in turn, modified by the surrounding fluid [1–3]. Air does not provide constant support against gravity and its properties vary at different temporal and spatial scales depending on turbulence. To save energy, flying animals therefore adjust timing, routes and flight modes to this turbulence [4], maximizing the advantage of horizontal and vertical air currents [5].

Soaring birds represent an extreme example of this adaptation. These large and heavy birds are particularly constrained in the use of active flapping flight, as the energetic cost of flight proportionally increases with size and weight [6]. They therefore use passive soaring-gliding flight, which is subsidized by the vertical air currents (uplifts) and may require as little energy as resting [7,8]. Body mass, wing loading and wing aspect ratio ultimately determine the cost of flapping flight, and with it a species' degree of dependence on uplifts [6,9–11]. This dependence becomes extreme in obligate soaring birds, which, due to their large size, can only fly in good uplift conditions, minimizing the use of flapping flight [12,13].

Uplifts originate from thermal convection (thermals) and/or mechanical sources (orographic uplift) [12,14]. Thermals originate from uneven heating of the earth's surface, with rapidly heated areas producing a gradient of temperature which promotes the formation of rising columns of warm air. Orographic uplifts, by contrast, result from the deflection of horizontal wind through topographic features, such as hills or ridges [12]. Thus, the occurrence of both thermal and orographic uplifts depends on a combination of local weather conditions (gradient of air temperature, wind speed and direction) and landscape features (land cover, slope inclination and aspect, roughness of the surface). The interaction of local weather conditions and landscape features thus characterizes a complex and turbulent atmospheric layer, where the spatial and temporal availability of uplifts constrains and shapes the movement patterns of soaring birds, from local scale to migratory routes [3,15–17].

Over the past decade, different models have been developed to investigate the relationship between soaring behaviour and aerial environment [8,18–21]. In many of these studies, the availability of uplifts was indirectly inferred using several weather parameters [22–25], but in recent years, these parameters have been replaced by thermal and orographic uplift potentials, as more direct estimators to quantify the probability of soaring [14,18–20,26–29]. However, some studies highlighted the inaptitude of these newly introduced variables as uplift estimators, because of the large amount of unexplained variance remaining when predicting soaring behaviour [18,19]. In fact, thermal and orographic uplift potentials are calculated based on different weather parameters [14,19], but because the uplift events are characterized by turbulences at fine spatio-temporal scale [12,19] it is challenging to predict their occurrences due to the limited spatio-temporal resolution of the available weather products.

In contrast to weather products, publicly available satellite data provide valuable static landscape information (such as land cover and elevation) at higher spatial resolution, which could be used to predict the occurrence of uplifts. Soaring birds need to locate uplifts in order to move across the landscape. Consequently, landscape features that influence uplift generation, might serve as visual cues to these birds, as they do for hang glider or paraglider pilots. By determining landscape features that birds use to locate uplifts, we may be able to predict those uplifts that are detectable and exploited by the birds.

Static landscape features alone could therefore potentially suffice in modelling the occurrence of uplifts, providing an answer to ‘where’, albeit not ‘when’, uplifts are likely to occur. Although the literature on the topic is scarce, some studies hinted at the role of static features in affecting the flight behaviour of different soaring species [25,28,30–33].

Here, we investigate to what extent static landscape features can represent the potential for generating uplifts. We explore this in an obligate soaring bird species, the white stork *Ciconia ciconia*, across the entire continent of Europe. We used first the locations of soaring and flapping behaviours of storks as an indication of the presence or absence of uplifts, and second their vertical speed as a proxy of uplift intensity. We then used only static features of the landscape to model and predict the spatial distribution of uplifts and their intensity across Europe. We also evaluated the effectiveness of these two static models by comparing their performances with the performances of two dynamic models, which included atmospheric uplift estimators used in previous studies. Finally, we explored the cost of flight (in terms of overall dynamic body acceleration) over the considered area, only based on the static landscape features. Under the assumption that soaring/flapping behaviour and vertical speed of the birds can be used as sensors of availability and intensity of uplifts, we predicted that (i) static features of the landscape (such as topography and land cover) can be used to predict the spatial availability of uplifts and to produce a static uplift suitability map at European scale; (ii) areas detected as suitable for uplifts during the first step can be further characterized in terms of uplift intensity likely to be produced in those areas; (iii) the resulting static uplift suitability map corresponds to the spatial distribution of the energetic costs of storks flying above this landscape, and thus portrays their static energy landscape.

## 2. Methods

### 2.1. Dataset

The GPS and tri-axial accelerometry (ACC) data used in the study were collected by the Max Planck Institute for Ornithology (see [34,35]) and are deposited in the Movebank Data Repository (http://dx.doi.org/10.5441/001/1.bj96m274 [36]).

**Figure 1.**
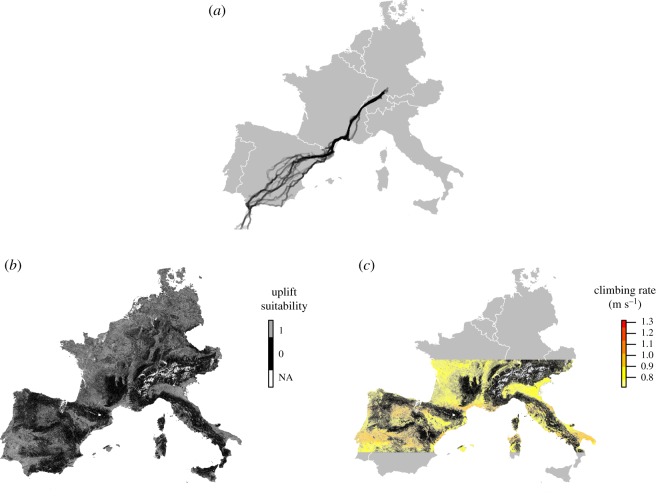
Spatial coverage of the white storks' migration routes, relative to the extent of the environmental layers included in the model. Black lines correspond to individual stork GPS trajectories (*a*). Static uplift prediction maps produced using the uplift suitability model (*b*) and the uplift intensity model (*c*), projected outside the geographical range of the training set. In (*b*), the colour scale corresponds to uplift suitability, as predicted by the uplift suitability model; grey indicates suitable and black unsuitable cells. White represents unclassified cells (containing missing values among the predictors). In (*c*), cells predicted as suitable are further characterized by the predicted uplift intensity values. Colour scale corresponds to vertical speed ranging from red (high) to light yellow (low). As in (*b*), in (*c*) black represents cells that are unsuitable for uplift and white indicates unclassified cells. Latitude values outside the range of the training set were excluded from the intensity model. The two prediction maps are available at https://dx.doi.org/10.17617/3.1u.

The animals were equipped as fledglings with high-resolution, solar GSM-GPS-ACC loggers (e-obs GmbH, Munich, Germany). The dataset includes 61 juvenile white storks (*Ciconia ciconia*) during their first migration (figure 1*a*). Because storks are diurnal, loggers provided one GPS location every 5 min between 2.00 and 20.00 GMT. If instantaneous ground speed was greater than 2 m s^−1^, bursts of high-resolution GPS locations (1 Hz) were recorded every 15 min for 120 or 300 s. In addition to the GPS locations, ACC was recorded every 10 min for a duration of 3.8 s at a sampling rate of 10.54 Hz (40 data points per axis). High-resolution GPS recordings were collected from August to September 2014.

### 2.2. Segmentation of the flight behaviour

#### 2.2.1. Soaring flight (from GPS)

We selected high-resolution GPS bursts with a duration of at least 120 s. For each location in the burst, we calculated vertical speed and turning angle. We applied our behavioural segmentation on track segments of 15 s (average duration of one complete soaring circle [34]). We calculated the average vertical speed and the absolute cumulative turning angle in these segments, and we used the expectation maximization binary clustering (EmbC) algorithm to discern the flight behaviours, introducing these two metrics as delimiters. The algorithm, implemented in the R package *EmbC* [37], efficiently detected changes in the flight behaviour, distinguishing a high turning angle (circular soaring) from two low turning angle clusters (linear flight). Based on the average vertical speed, we further differentiated the linear flight segments into gliding (linear descending flights) and linear soaring (linear ascending flights). Each 15 s segment along the animal trajectory was individually assigned to one of the behavioural classes based on its specific parameters. We applied a smoother to avoid abrupt and unnatural behavioural changes from one segment to the next along the same trajectory. Our smoother worked as a moving window: each segment assigned to a different behaviour relative to its closest neighbours was reclassified to match the modal value of two segments before and after the considered segment.

**Figure 2.**
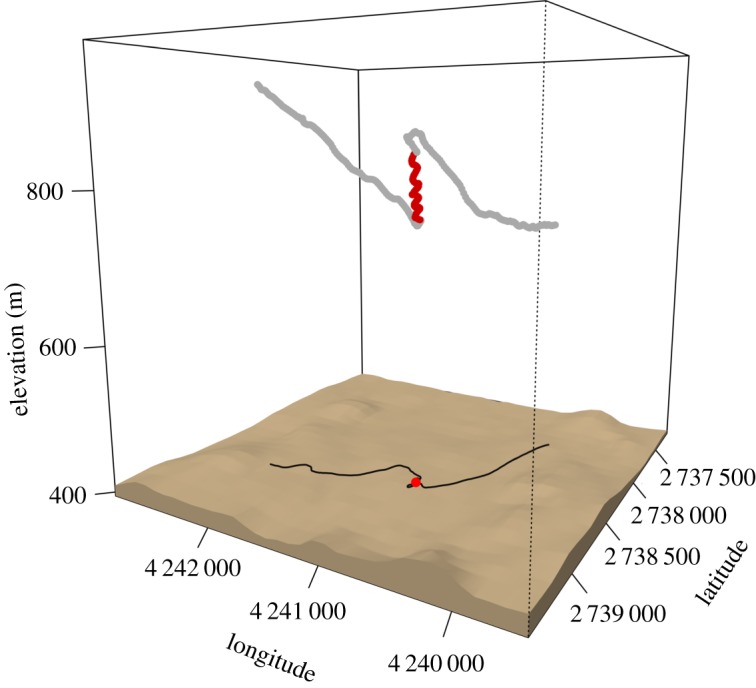
Example of behavioural segmentation based on the GPS data of one stork. The figure shows the classified three-dimensional trajectory after smoothing; the red segment was identified as soaring flight, grey corresponds to gliding flight. Data for plotting the surface are provided by the EU-DEM. The black line and the red point on the ground represent the two-dimensional projection of the trajectory and the centroid of the soaring segment, respectively.

Given the high resolution of the GPS data, we could visually inspect and confirm the results of the segmentation using three-dimensional plots (figure 2). We then investigated the different classified behaviours in terms of their flight parameters, such as ground speed or vertical speed (electronic supplementary material, S1, figure S1.1).

In the subsequent steps, we wanted to contrast the use of active versus passive flight, focusing on the dichotomy soaring/flapping. We therefore did not differentiate between circular and linear soaring (both classified as soaring), and we excluded gliding segments, as they are not considered as an alternative to soaring (like flapping) but rather as its consequence [12]. In these analyses, we considered for each individual only soaring segments with a duration longer than 30 s, and treated consecutive soaring segments as different units only when separated by at least 60 s. The location of each soaring segment was defined by its centroid (mean longitude and latitude).

#### 2.2.2. Flapping flight (from tri-axial accelerometry)

We interpolated the spatial location of each ACC burst based on the closest GPS locations using the R package *move* [38]. We associated each ACC burst with the height above ground corresponding to the GPS location closest in time (less than 30 s difference). The height above ground was calculated by subtracting the ground elevation value from the height above the ellipsoid.

We used ACC values to identify bursts of active flight behaviour (flapping flight). Specifically, we used overall dynamic body acceleration (ODBA), already shown to be a good proxy for energy expenditure in soaring birds [8,39]. We quantified ODBA and dynamic body acceleration (DBA) on the *z*-axis following Wilson *et al.* [40] and calculated mean, sum and standard deviation, of these two variables per burst. We then used k-means clustering to categorize the bursts into three main behavioural classes based on the amount of activity recorded: least active, intermediate active and most active (electronic supplementary material, S1, figure S1.2). Within the bursts of highest activity, we wanted to isolate only the flapping behaviour marking the absence of uplifts (and to exclude, for instance, the flapping associated with taking off); we thus applied a height threshold of 100 m above ground to select our flapping locations, assuming that above this height the birds were using flapping flight only in response to the absence of uplifts.

The two flight behaviours were classified based on data collected with different instruments running on different sampling schedules (GPS for soaring and ACC for flapping). Therefore, the amount of soaring to flapping locations is not directly related to the amount of time storks spent on each flight behaviour. We thus compared the amount of time spent soaring relative to the total duration of the classified GPS segments, and the amount of time spent flapping relative to the total duration of the classified ACC bursts.

### 2.3. Environmental variables and modelling frameworks

#### 2.3.1. Static predictors

We characterized the static components of the landscape in terms of elevation (digital elevation model, EU-DEM 2013), terrain unevenness (calculated as both topographic position index and roughness), unevenness in the slope (steepness of a terrain feature), aspect (compass direction faced by a slope), aspect unevenness, land cover (normalized difference vegetation index, NDVI, obtained for 2014), land use (CORINE Land Cover; CLC 2012) and presence of anthropogenic infrastructures (Global Urban Footprint, 2011). All raster layers are publicly available (electronic supplementary material, S2, table S2.1). The lowest spatial resolution was 100 m (from the CLC 2012 layer), thus we averaged cell values of higher resolution layers to match a 100 m grid. The spatial extent of the raster layers covered the southwest European countries that enclose the distribution of the storks' dataset. All the environmental layers listed above were included as predictors in our statistical models after verifying the absence of multicollinearity.

#### 2.3.2. Dynamic predictors

We chose to include thermal and orographic uplift potentials in our analysis as atmospheric uplift estimators [14,18,20,26,27]. The calculation of the thermal uplift potential is based on weather data from the European Centre for Medium-range Weather Forecast Global Atmospheric Reanalysis (ECMWF) following Bohrer *et al.* [19]. The calculation of the orographic uplift potential uses ECMWF weather data and elevation from the ASTER Global Digital Elevation Model. Both thermal and orographic uplift potential are available in Movebank with a spatial resolution of 0.75° and a temporal resolution of 6 h. We associated them to our tracking data by using the Env-DATA Track Annotation service [41].

### 2.4. Modelling framework

We organized the environmental predictors in three groups, each defining a different modelling framework:

1. Static model: including exclusively static environmental predictors;
2. Dynamic model: including exclusively thermal and the orographic uplift potentials;
3. Combined model: including both static and dynamic predictors.

We used these sets of predictors for both the uplift suitability and the uplift intensity models.

### 2.5. Uplift suitability model

We used a random forest algorithm to model the effect of the three sets of environmental predictors on the occurrence of soaring (presence of uplifts) and flapping flight (absence of uplifts), using these contrasting behaviours as binary response variable. The algorithm is implemented in the R package *randomForest* [42]. We manipulated the ratio between presences and absences (prevalence) and tested its effect on the model performance (see electronic supplementary material, S3). In our analysis, we included all the available data with their original (unmanipulated) prevalence values. Using regression trees, we trained each of the three models (static, dynamic and combined) with 90% of the dataset, and tested them with the remaining randomly selected 10%. The data partitioning was repeated so that each of the three models was run ten times. To evaluate and compare the predictive performance of the three models, we considered the following accuracy measures: (i) area under the curve (AUC) of the receiver operating characteristic (ROC); (ii) sensitivity, proportion of soaring locations correctly classified; (iii) specificity, proportion of flapping locations correctly classified [43]; (iv) true skill statistics (TSS: 1−max(sensitivity + specificity)) [44]. The contribution of each environmental variable to the final prediction was evaluated using the decrease in accuracy (increase in mean standard error) and the increase in node purity (decrease in residual sum of squares).

Next, we produced a large-scale uplift suitability map based on the static uplift suitability model. Random forest, like other machine learning algorithms, is quite unreliable when extrapolating outside the range of the predictors' values provided for training. We thus omitted (set to null) all raster cells containing environmental values outside that range, and then used each of ten runs of the static uplift suitability model to predict the uplift suitability over the area of these manipulated raster layers. The final raster prediction was derived from the pixel average of the ten predicted layers and classified into a binary map using the threshold that maximized the TSS value [43].

The only temporally related environmental variable in our model was NDVI from the year 2014; this allowed us to produce an uplift suitability map for 2014.

### 2.6. Uplift intensity model

We explored the relationship between the three sets of predictors (static, dynamic and combined) and uplift intensity, additionally including latitude among the static predictors. We used the birds' vertical speed as a proxy of uplift intensity (vertical rate of air within a thermal), assuming a higher vertical speed to indicate stronger uplift conditions.

As in the previous analysis, we considered only high-resolution GPS bursts. The vertical speed in this dataset included both negative (gliding) and positive values (soaring). We examined only the positive values (vertical speed greater than 0), because we wanted to predict uplift intensity in areas already classified as suitable by the uplift suitability model. We associated the positive vertical speed values of all individuals with their location and averaged them in a 100 × 100 m grid to match the spatial resolution of the environmental raster layers. After averaging, each cell contained a value representing the average vertical speed of all individuals during the complete temporal range in that cell. We then removed average vertical speed values exceeding the 99.97 percentile, obtaining 76 383 observations.

We used a generalized additive model (GAM) to model uplift intensity (average vertical speed) as a function of the three sets of environmental predictors to accommodate nonlinear relationships between predictors and response variable. We square-root transformed vertical speed to meet the assumptions of a Gaussian distribution of the residuals. Among the predictors, aspect was included as cyclic cubic regression spline smooth term; NDVI, elevation (DEM), roughness and latitude were included as thin plate regression spline smooth terms, given their nonlinear relationship with the response variable. We rasterized the values of thermal and orographic uplift potentials included in the dynamic and combined models to match the 100 × 100 m grid of the response variable, and included them in the models as parametric coefficients.

We used the static uplift intensity model to produce a map of uplift intensity, and enrich the binary information provided by the uplift suitability model in areas that were predicted as suitable. Raster cells containing environmental values outside the range included in the dataset were omitted; because latitude was included as predictor, the latitudinal range of the uplift intensity map was restricted to the latitudinal range of the dataset.

The models were run in R using the package *mgcv* [45]. We compared the performances of the models based on the variance explained and the AIC (Akaike Information Criterion). The relative importance of the different predictors was evaluated comparing the AIC of models containing different combinations of these predictors, computed separately for parametric coefficients and the smooth terms.

### 2.7. Static energy landscape

We quantified the relationship between the availability of uplifts along the storks' migratory routes and the energy spent travelling along these routes. We could thus test if the static uplift suitability map produced in the previous step could convey information regarding the energetic cost of travelling across the landscape. We calculated the daily energy expenditure considering only ACC data collected when the animals were flying (height above ground > 100 m), with GPS location and ACC burst matching in time. We then calculated the mean ODBA per day along the path of each individual. The uplift suitability map was used to extract the predicted probability of uplift at the locations of the ACC bursts. We then averaged these probability values to obtain the mean daily uplift suitability, for each individual, along its migratory path (only average values computed from at least five observations were included in the model). We fitted a linear mixed effect regression model to the mean daily energy expenditure (ODBA) as a function of predicted mean daily uplift suitability. The model tested the relationship between daily uplift suitability predictions and daily ODBA based on 823 observations of 59 individuals, accounting for individual differences which were included as random effects in the model. ODBA was square-root transformed.

The importance of the predictor in explaining the daily energy expenditure was assessed comparing the AIC of the model with the respective null model. For the analysis, we used the R package *lme4* [46]*.*

## 3. Results

### 3.1. Segmentation of the flight behaviour

We identified the location of soaring and flapping flights as proxies to detect the presence and absence of uplifts. Based on the GPS data of all individuals, we classified over 748 h of flight, of which the storks spent 297.6 h with circular and 83.5 h with linear soaring. The proportion of time spent soaring corresponded to 0.51 of their flight time (381.1 h) (electronic supplementary material S1, figure S1.3a); this proportion was similar between the 59 individuals (0.52 ± 0.07 (mean proportion ± s.d. per individual)). From the ACC data, we classified 24.3 h of flight, of which 1.3 h was spent flapping (electronic supplementary material, S1, figure S1.3b). Among all individuals, the proportion of time spent flapping corresponded to 0.05 (0.07 ± 0.05 per individual). The final dataset consisted of a total of 16 840 observations of presences and absences of uplift (15 608 soaring events marking presences and 1232 flapping events marking absences).

### 3.2. Uplift suitability model

We used multiple environmental predictors to model and predict the spatial distribution of uplifts (presence and absence data). We organized the predictors in three different modelling frameworks (static, dynamic and combined, see Methods) that we then compared in terms of predictive accuracy.

**Figure 3.**
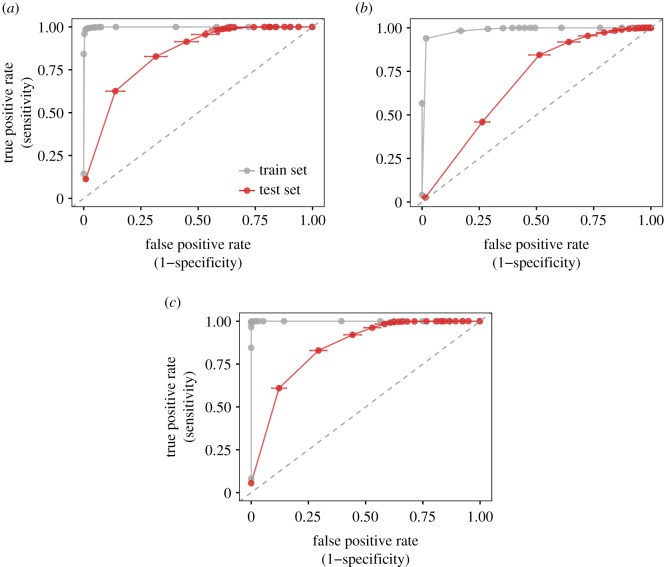
ROC curves of the three uplift suitability models: static (*a*), dynamic (*b*) and combined (*c*). The area under the curve (AUC) represents the accuracy of the model. The accuracy was measured on both the training set (grey solid line) and the test set (red line). The dashed line represents a model whose accuracy is comparable to random (AUC = 0.5). Sensitivity and commission rate values were averaged across the 10 runs of each models (solid dots), and the error bars show their standard deviations.

We averaged the accuracy measures of the three uplift suitability models across 10 cross-validations. The combined model (static and dynamic features) best predicted the independent test set (AUC of 0.86 ± 0.02 (mean ± s.d.)), followed by the static (AUC of 0.85 ± 0.02) and the dynamic (AUC of 0.70 ± 0.02) models (figure 3). The overall accuracy was high in all models, but both models including static variables (the static and combined models) outperformed the model based only on dynamic predictors.

We then compared the ability of the models to discriminate presences and absences. The three models returned a similarly high proportion of correctly classified soaring locations (sensitivity). They differed, however, in terms of number of correctly classified flapping locations (specificity). Again, the combined and the static model outperformed the dynamic model. To define the value of sensitivity and specificity, we used a threshold that maximized the TSS value, corresponding to 0.9 in all models. At this threshold, the static model showed a sensitivity of 0.83 ± 0.01 and a specificity of 0.69 ± 0.05. The complete output of the three models can be found in electronic supplementary material, S3, table S3.1.

In the static model, DEM, roughness (topographic heterogeneity) and NDVI contributed most to the model prediction. In the dynamic model, including only the two atmospheric uplift estimators, the thermal uplift potential contributed to the prediction more than the orographic potential; in the combined model, elevation, roughness and thermal uplift potential contributed most to the model (for more details see electronic supplementary material, S3).

Using the static uplift suitability model, we produced a map of uplift suitability covering the extent of the environmental layers (figure 1*b*). We classified an area of about 193 million km^2^, of which over 81 million km^2^ was predicted as suitable for uplifts (42% of the total area).

### 3.3. Uplift intensity model

We then used the uplift intensity to characterize those areas identified as suitable for uplifts by the static suitability model. We used the vertical speed of the birds while soaring as a proxy for uplift intensity, and we explored the relationship between uplift intensity and the three groups of environmental predictors (static, dynamic and combined).

All three models explained very little of the total variance in vertical speed. However, here too, the combination of static and dynamic variables provided the best predictive performance (Adj.*R*^2^ = 0.03 and AIC = 46 575.00 for the static model; Adj.*R*^2^ = 0.03 and AIC = 49 636.88 for the dynamic model; Adj.*R*^2^ = 0.08 and AIC = 42 003.95 for the combined model). Although in GAMs Adj.R^2^ values cannot be directly compared due to the changing degrees of freedoms caused by the use of smooth terms [47], the difference in the AIC value among the three models supports the best performances of the combined model. Among the parametric predictors, the categories ‘water bodies’, ‘dumps’, ‘urban areas’ and ‘wetlands’ negatively affected uplift intensity (‘bare soil’ served as a reference), whereas thermal and orographic uplift potentials (included in the dynamic and combined models) positively affected uplift intensity (electronic supplementary material, S4, table S4.1). Aspect, NDVI, DEM, roughness and latitude were included in the models as smooth terms, given their nonlinear relationship with the response variable. Based on AIC, all these predictors contributed to explain uplift intensity. Specifically, uplift intensity was positively affected by lower latitude values, higher elevations (DEM > 2000 m), NDVI corresponding to bare soils or sparsely vegetated areas (between 0 and 0.4) and slope orientation towards SW-W (aspect between 200° and 300°) (electronic supplementary material, S4, table S4.1 and figure S4.1). Using the static intensity model, we could further characterize our uplift suitability map by predicting uplift intensity in cells already predicted to be suitable for uplifts (based on the static suitability model) (figure 1*c*).

### 3.4. Static energy landscape

**Figure 4.**
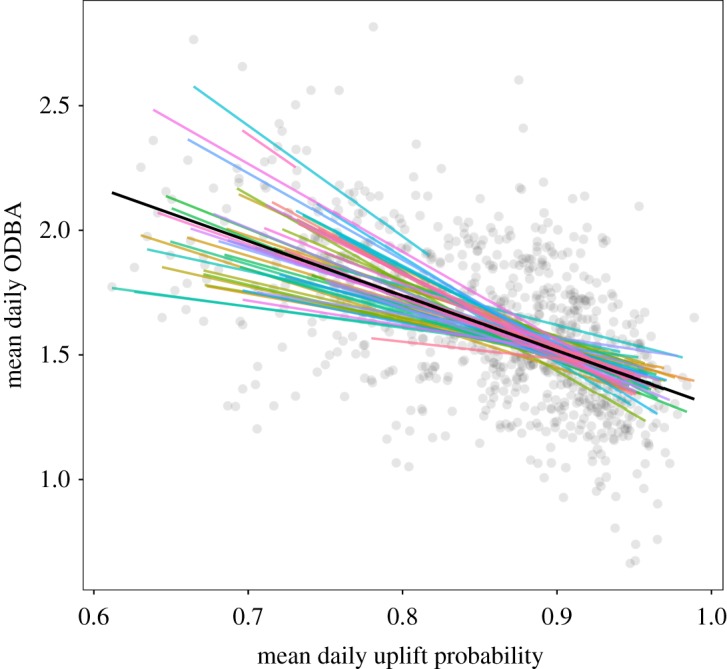
Mean daily ODBA along the storks’ routes as a function of the mean daily uplift suitability along those routes, as predicted by the static uplift suitability model. Coloured lines represent the different individuals accounted for in the linear mixed model. Grey points represent all observations included in the model.

Finally, we quantified the relationship between the availability of uplifts along the storks' migratory routes and the energy spent travelling along these routes, to test if the static maps produced in the previous steps could convey information regarding the energetic cost of travelling across the landscape. As all uplift intensity models performed poorly in predicting the intensity of uplifts, only the uplift suitability model was included in this step. We used a linear mixed effect regression model to evaluate the role of the static uplift suitability model in conveying information about the energy expenditure of the birds (measured as daily ODBA). A negative correlation between the daily uplift suitability and the daily ODBA indicated that the birds spent more energy when flying over areas less suitable for uplifts (ODBA = −0.67 ± 0.07 (estimate ± s.e.)) (figure 4). The AIC of the models was lower compared to that of the respective null model (ΔAIC uplift suitability model = −54.67) (electronic supplementary material, S5, table S5.1).

## 4. Discussion

Static features of the landscape proved to be highly effective in identifying areas suitable for uplifts. However, neither static nor dynamic variables could predict the intensity of uplifts occurring in those areas. The uplift suitability predicted along the birds' migratory route using only static features, showed a clear negative relationship with the ODBA of individuals flying over those areas, indicating that birds encountering fewer uplifts along their routes experienced higher energy expenditures. This overall result validates the reliability of our static uplift suitability model, and suggests the existence of a mechanistic relationship between static landscape and energy expenditure of flying animals. We therefore propose that the static uplift availability map produced with our model corresponds to the birds' cost of transport across the landscape and can thus be considered a representation of the static energy landscape of these birds.

The possibility to describe the cost of transport in a dynamic aerial environment, only based on static features of the landscape, supports the idea that the structure of the landscape at different spatial scales could be considered as the ultimate cause for the uplifts to occur. For instance, a specific topography could represent a necessary (but not sufficient) condition of uplift occurrence, or, in other words, the potential of the landscape to produce uplifts. By contrast, local weather conditions that interact with a specific landscape, could be considered as the proximate cause for the occurrence of the uplift, which can define, given a suitable landscape, the temporal scale at which the uplift will, in fact, exist.

In the case of the uplift intensity, even though models including static features performed slightly better than those including only dynamic variables, the large amount of unexplained variance in all models suggested that neither static nor dynamic environmental variables were good predictors for uplift intensity. In our models, we used the birds' vertical speed as a proxy for uplift intensity. But birds' vertical speed is not only affected by uplift intensity. Their relationship is modulated by the aerodynamic performance of the bird (including wing morphology), and also by its social interactions and motivation. The ability to adjust the vertical speed within a thermal requires experience [48,49]. The storks included in our study were all juveniles during their first migration, but even among individuals of the same age, individual differences in flight performances exist, also in relation to the role of the individual within the group (leader or follower) [35]. The vertical speed of the birds might also be affected by their internal motivation to move (foraging versus migrating). During migration, birds are expected to maximize their vertical speed and travelled distance, whereas while foraging they might adopt different strategies, for instance attempting to maintain lower altitudes [12], which could explain the negative effect of land use categories such as dumps or pastures in the uplift intensity models. All these aspects could have affected the observed vertical speed of the birds and thus caused the inconsistent relationship between uplift intensity and environmental variables in our models. The spatial and temporal scales at which the uplift phenomenon occurs might have also contributed to this inconsistency. Uplifts are a turbulent and unpredictable phenomenon and they can occur at very small scale [5] as in the extreme cases of lifts produced by lines of buildings or flared methane vents [20,50,51]. The intensity of an uplift, more than the presence of an uplift, is strongly influenced by wind speed, wind direction and temperature, and thus more subject to the temporal and spatial variation of these dynamic variables.

The inadequacy of the spatio-temporal resolution of dynamic uplift estimators is not new [18], and the coarse resolution of the atmospheric data could also explain why all models including only dynamic variables performed worse than those including static variables alone, in predicting both uplift availability and uplift intensity. Nevertheless, the effect of some of the static variables included in our uplift intensity models hinted at a dependence of the uplift intensity (as in the case of the uplift availability) on the static landscape structure. This is the case, for instance, of the negative effect of water bodies, and the positive contribution of higher elevations and NDVI values corresponding to barren soils, on the uplift intensity. Also, lower latitude values positively affected uplift intensity; this result suggests a stronger thermal activity at lower latitudes, but could also indicate that young storks improved their flight performance along the route.

The static landscape features used to produce our static uplift maps are definitely not exhaustive to describe the complex fluid medium in which flying animals move, but they could represent a sufficient and efficient proxy (in terms of computational simplification) in areas and seasons where weather conditions are rather stable. The prediction maps produced by our static models are based on data from one species collected during one migratory season, but the same models could be extended to multiple soaring species and different seasons in order to generalize predictions. Such prediction maps could be used as base layers for further movement ecology analyses, and combining them with dynamic variables could provide a more accurate description of the energy available at a specific moment.

Static energy landscapes can also direct our attention to the vulnerability of flying animals to changes happening at the ground level. Anthropogenic changes in the landscape, such as deforestation, construction of wind farms and powerlines, but also roads, lines of buildings and tree rows, irrigation, or mining, could all be affecting the atmospheric environment, at a finer scale than the available weather products could possibly detect. The tight dependence of soaring birds on uplift conditions makes them particularly sensitive to changes in the landscape [19,52], in particular to anthropogenic infrastructures [10,53,54]. Our study suggests that these small changes in the landscape could affect the energy expenditure of these animals, and potentially their cost of transport over time. The static structure of the landscape and the energetic implications of changes happening on the ground should therefore be taken into account when investigating movement at larger scale, such as migratory flyways and population connectivity, and when evaluating the impact of anthropogenic infrastructure. Future studies should also focus on the interplay between vertical speed, uplift intensity and environment. By disentangling the various factors affecting this relationship, we could not only predict, based on the animal behaviour and the landscape, the quantity (availability) but also the quality (intensity) of the uplifts, and we could provide a more accurate estimation of the energetic cost of movement across the landscape.

## Supplementary Material

Supplementary text, figures and tables

## Data accessibility

The data used for this study are deposited in the Movebank Data Repository: (http://dx.doi.org/10.5441/001/1.bj96m274) [36].
