# Supplementary material for: Static landscape features predict uplift locations for soaring birds across Europe
Source: R Soc Open Sci. 2019 Jan 16;6(1):181440. doi: 10.1098/rsos.181440 (PMC6366234; doi:10.1098/rsos.181440)
Supplement: Supplementary text, figures and tables [file rsos181440supp1.pdf]

Static landscape features predict uplift locations for soaring birds across Europe

Martina Scacco, Andrea Flack, Olivier Duriez, Martin Wikelski, Kamran Safi

SUPPLEMENTARY MATERIAL

List of content

S1 – Segmentation of flight behaviour. Containing supporting figures (Fig. S1.1, Fig. S1.2 and Fig. S1.3).

S2 – Environmental variables. Containing more details about the procedure we used to handle the static environmental layers; Tab. S2.1 table with environmental variables sources; Tab. S2.2 table containing the old legend and the reclassified legend of the Corine Land Cover classes.

S3 – Uplift suitability model. Containing additional figures (Fig. S3.1, S3.2, S3.3) and tables (Tab. S3.1) supporting the results. It also contains the detailed procedure and results of the random forest tuning and prevalence test.

S4 – Uplift intensity model. Containing the output of the three GAMs (Tab. S4.1) and supporting figure (Fig. S4.1).

S5 - Static energy landscape. Containing the output table of the linear mixed model (Tab. S5.1).

S1 – Segmentation of the flight behaviour

Supporting figures for segmentation

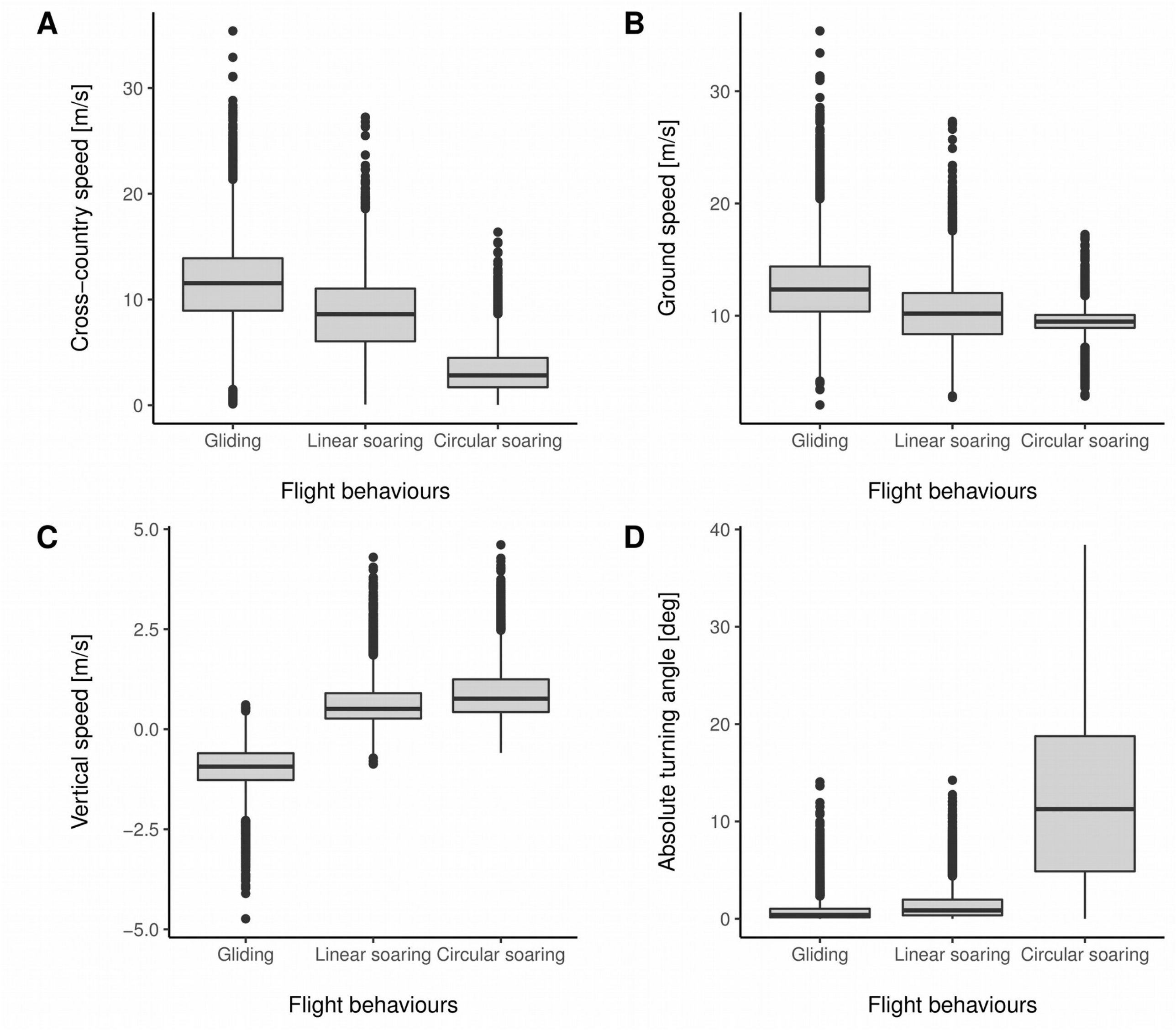

Figure S1.1 - Characterization of the different flight behavioural classes according to different flight parameters: cross-country speed (A), ground speed (B), vertical speed (C) and absolute turning angle (D).

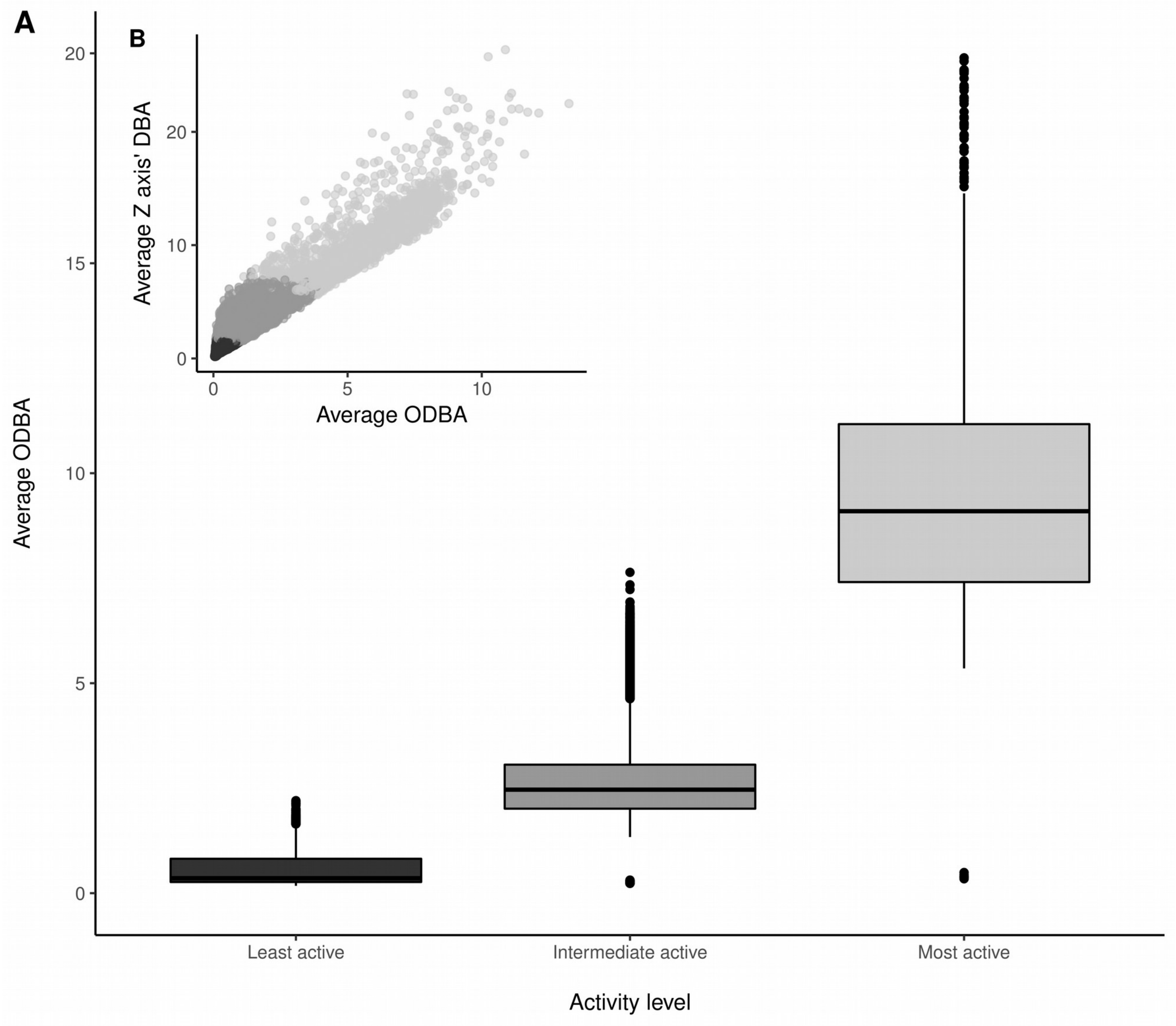

Figure S1.2 - Example of segmentation of the flight behaviour detected from the ACC data of one individual of white stork. Figure (A) shows the different empirical values of the average ODBA among the three activity levels. Figure (B) shows how the three activity categories are different in terms of dynamic body acceleration measured on the three ACC axes (plot's x axis) and on the Z axis (plot's y axis).

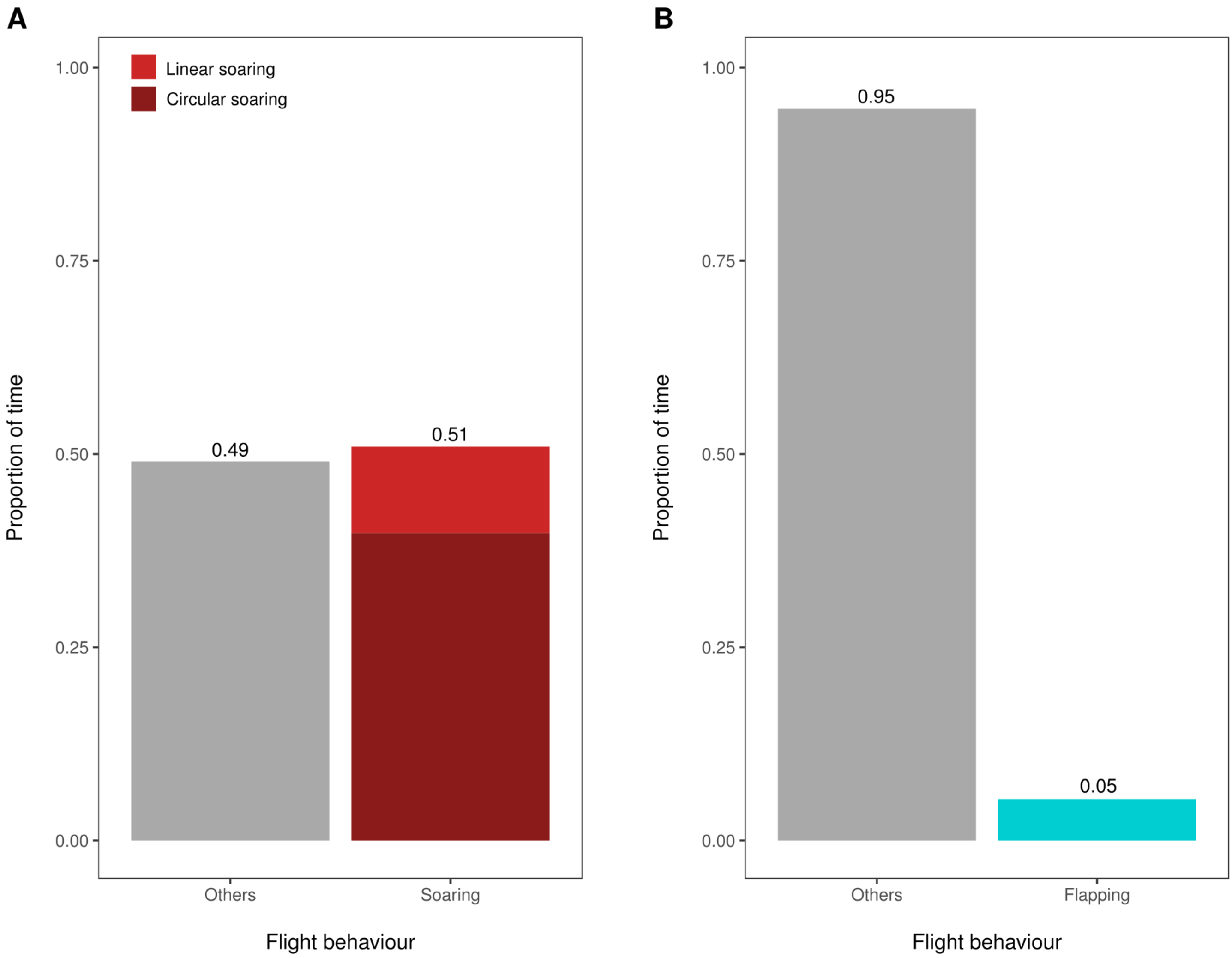

Figure S1.3 - Time allocation among flight behaviours. The barplots show the proportion of time white storks spent performing different flight behaviours (proportions obtained by cumulating the time spent by all individuals on the different behaviours). (A) Proportion of time (classified using GPS locations) spent soaring (circular or linear soaring, red bar) or using other flight types (grey bar) calculated relative to the duration of all classified GPS segments (748 h). (B) Proportion of time (classified using ACC recordings) spent flapping (blue bar) or using other flight types (grey bar) relative to the duration of all classified ACC bursts (24.3 h). The information shown in figures (A) and (B) cannot be directly compared due to the different sampling schedules of GPS and ACC.

S2 – Environmental variables

Static environmental variables

For descriptive purposes we group the environmental layers in two categories - surface features and land cover. All environmental layers are publicly available (Tab. S2.1).

Surface features. In order to characterize the surface features we used the publicly available elevation map EU-DEM (based on SRTM and ASTER Global Digital Elevation Model) [1] and we computed slope, aspect and roughness (topographic heterogeneity) using the R package *raster* [2]. The native spatial granularity of the elevation map is 1 arcsec (about 25 m near the Equator) but we aggregated the raster cells to match the 100 m resolution of the land cover map. Slope and aspect were computed according to Horn [3]. The roughness was calculated as the difference between the maximum and the minimum value of a cell and its 8 surrounding cells. Unevenness in the aspect, the slope and the elevation (this last one also called Topographic Position Index) were computed as the difference between the value of a cell and the mean value of its 8 surrounding cells. Highly correlated layers were excluded from the model to avoid multicollinearity (this was the case of the slope because of the high correlation with the roughness).

Land cover. We characterized the land cover using the Normalized Difference Vegetation Index (NDVI), CORINE Land Cover categories (CLC) and the Global Urban Footprint (GUF). The NDVI product is available as Spectral Indices product of Landsat 7 [4] with a spatial granularity of 30 m; the raster cells were resized to 100 m. We computed a summer (from June 1st to September 30th) NDVI composite for 2014 to match the temporal resolution of our tracking data. For the composite we extracted the higher monthly NDVI value of each cell and we averaged the resulting maximum monthly values. Extracting the maximum monthly value instead of the average value allowed us to avoid low values of NDVI that could be associated with errors in the

cloud cover mask of the Landsat NDVI product. The CORINE Land Cover 2012 is available from the European Environmental Agency [5] with a native spatial resolution of 100 m. We used the level 3 categories with few modifications (Table S2.2). The Global Urban Footprint is a binary thematic map with values of 1 for built-up areas (man-made building structures) and 0 for non-built-up areas. The dataset is available with a native spatial resolution of 0.4 arc seconds (about 12 m near the Equator) [6]. We resized the raster cells to 100 m computing the mean for each 9 by 9 cells (proportion of built-up areas/100 m cell).

Table S2.1 - Environmental data sources.

Environmental layer	source
Digital elevation model (DEM)	EU-DEM (based on SRTM and ASTER Global Digital Elevation Model) from European Environmental Agency [1].
Roughness	Derived from DEM
Topographic Position Index	Derived from DEM
Slope	Derived from DEM
Slope unevenness	Derived from Slope
Aspect	Derived from DEM
Aspect unevenness	Derived from Aspect
Normalized Difference Vegetation Index (NDVI)	Landsat 7 Spectral Indices [4]. Available from U.S. Geological Service Bulk Download [9]. Data period June-September 2014.
CORINE Land Cover (CLC)	CLC 2012 from European Environmental Agency [5].
Global urban footprint (GUF)	Produced by Deutschen Zentrums für Luft und Raumfahrt, 2011 [6].
Thermal uplift potential	Movebank.org – Env-Data annotation service [7] based on ECMWF weather reanalysis, computed following Bohrer *et al.* [8].
Orographic uplift potential	Movebank.org – Env-Data annotation service [7] based on ECMWF weather reanalysis and ASTER DEM, computed following Bohrer *et al.* [8].

Table S2.2: Original and reclassified legend of the CORINE Land Cover categories.

Reclassified categories	Reclassified code	CLC 2012 Legend
Dumps	1	8
Artificial vegetated areas	2	10-11
Arable lands	3	12-14
Permanent crops	4	15-17
Pastures	5	18
Heterogeneous agricultural areas	6	19-22
Forest	7	23-25
Shrubs	8	26-29
Glaciers, snow	9	34
Inland wetlands, marshes	10	35-36
Marine wetlands, salines, inter-tidal flats	11	37-39
Water courses, rivers	12	40
Water bodies, lakes	13	41
Coastal lagoons, estuaries	14	42-43
Sea, ocean	15	44
Urban areas	16	1-6,9
Bare soil	17	7,30-33
No data or unclassified	NA	48-50,255

S3 – Uplift suitability model

Model tuning and evaluation

Different parameter values can be modified by the user in order to tune the random forest algorithm and improve its performances (such as number of variables chosen at each split, forest size and tree depth). In addition, in the field of species distribution modelling, the proportion of data belonging to different classes in the dataset used to train the model has an important effect on different accuracy measures on the model output of different machine learning algorithms [10,11]. For these reasons, we tuned the static and the dynamic landscape models, choosing the optimal values of mtry (number of variables chosen at each split) with respect to Out-of-Bag error estimate, using the package's inbuilt function *tuneRF*. Additionally, we tried different values of ratio soaring to flapping locations (named hereafter prevalence, for similarity with the definition used in species distribution modelling).

The role of prevalence on the model performances is controversial. Different studies suggested that certain accuracy measures (such as kappa) are sensitive to prevalence and that prevalence should be taken into account when evaluating the model [12,13], but few unclear suggestions have been made about whether the number of presences and absences should be manipulated in order to maximize the model performances [10,11,13]. This lack of clarity is probably due to multiple reasons, such as the algorithm used, the difference between using real absences or pseudo-absences, the difficulty to differentiate between biases in the measures used to evaluate the performance and biases in the performances itself, and finally to the uncertain effect of data manipulation on the ecological interpretation of the result (for instance when dealing with rare and specialist species versus abundant and generalist ones, and the need to compare the predicted prevalence with the observed prevalence). For this reasons we decided not to manipulate the prevalence in our analysis but to perform a

prevalence manipulation test. The prevalence manipulation test was performed on 100 trees. Before running the model with different prevalence values, the complete dataset was randomly partitioned in test set (20% of the data) and train set (80% of the data), separately for soaring (presences) and flapping (absences) locations in order to maintain the same ratio of presences to absences in both the original dataset and the test set; from the train set we then manipulated the number of presences to meet the values of prevalence (n. presences/n. absences) we wanted to test. The same data partitioning procedure was repeated 10 times. The data partition used to test the model was separated from the training set before manipulating the prevalence in the training set; in this way we could ensure a constant size for the test set during the evaluation of each prevalence value. During the models' evaluation we considered the following accuracy measures: AUC or area under the ROC curve, sensitivity and specificity; sensitivity and specificity are threshold dependent measures and the chosen values correspond to a threshold that maximize the sum of specificity and sensitivity (True Skill Statistics or TSS).

The results of the tuning procedure showed an increase in the model accuracy (AUC and TSS) with increasing prevalence. Sensitivity and specificity showed variable values at different values of prevalence, but they both showed a slight positive trend and the difference between the two slightly decreased, with increasing prevalence (Fig. S3.1). In contrast, the threshold that maximized the TSS increased, being around 0.5 with a prevalence equal to 2, and around 0.9 with a prevalence of 12, which is in agreement with the fact that the best performances in our model were associated with high thresholds. This didn't affect our model accuracy, but its robustness. In fact, all models including the original presence/absence ratio (12.66), the threshold at which the number of flapping locations correctly classified matched the number of soaring locations correctly classified was really close to 1, between 0.9 and 0.95; a threshold

of 0.9 favoured the identification of soaring (with higher commission rate) whereas a threshold of 0.95 favoured the identification of flapping (with higher omission rate). This means that this model prediction, although accurate, can be considered sensitive compared to a prediction obtained with a lower threshold, and a small change in the threshold leads to really different predicted results. For this reason, if the sample size of the least represented class allows it, we recommend the use of balanced classes in species distribution and habitat suitability modelling, by randomly subsampling the more represented class to about twice the size of the least represented class, to be able to choose a more centered threshold and increase model robustness.

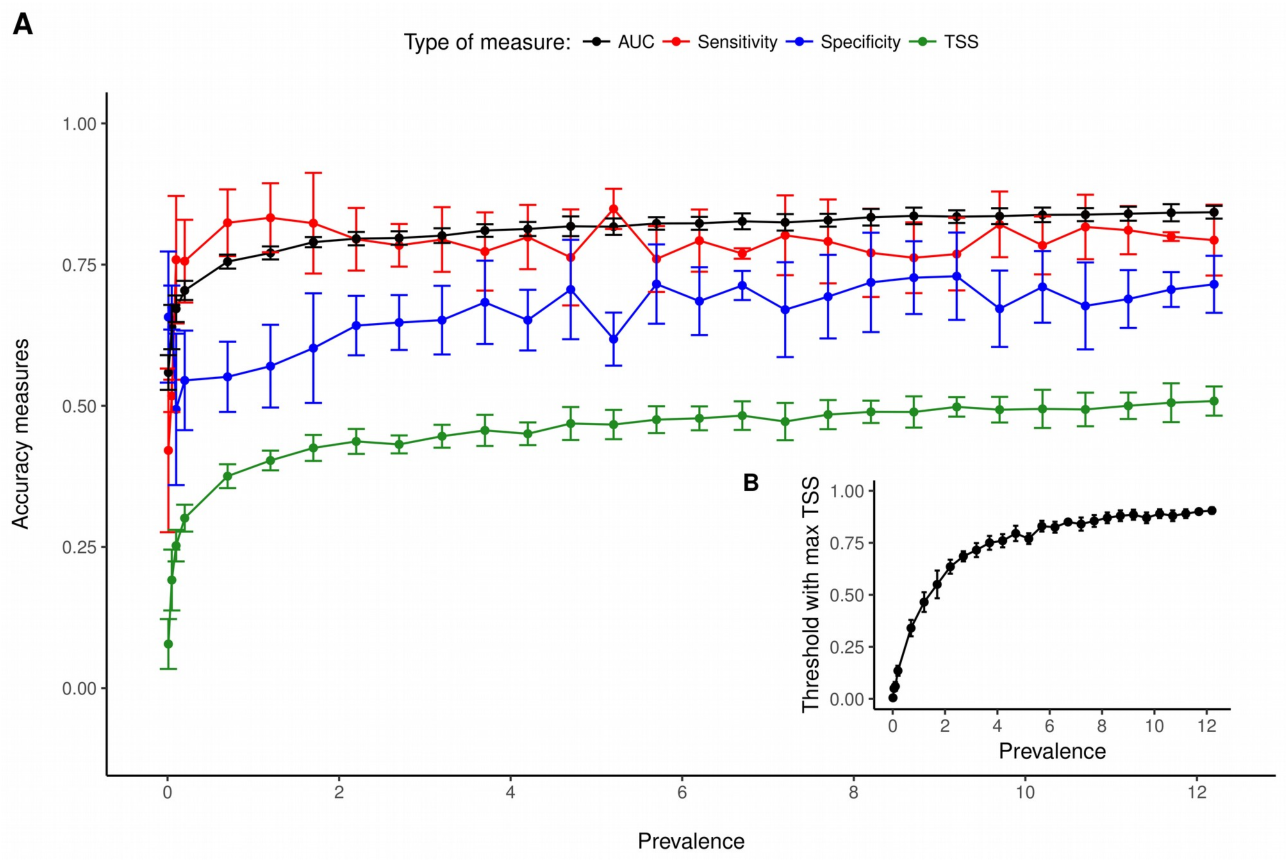

Figure S3.1 - Prevalence test. Effect of different prevalence values (ratio soaring to flapping locations) on different accuracy measures (A), and on the threshold that has to be chosen in order to maximize True Skill Statistics, calculated as (Sensitivity + Specificity) – 1 (B).

Table S3.1 - Random forest evaluation of the three uplift suitability models, based on the test set and averaged across the ten cross-validations.

Table S3.1 A - Static uplift suitability model, based only on static features (AUC±sd = 0.851±0.022).

Threshold	Sensitivity	Specificity	Commission Error	Omission Error	TSS
0	1 (0)	0.001 (0.003)	0.999 (0.003)	0 (0)	0.001 (0.003)
0.05	1 (0)	0.061 (0.022)	0.939 (0.022)	0 (0)	0.061 (0.022)
0.1	1 (0)	0.097 (0.033)	0.903 (0.033)	0 (0)	0.097 (0.033)
0.15	1 (0)	0.122 (0.025)	0.878 (0.025)	0 (0)	0.122 (0.025)
0.2	1 (0)	0.162 (0.044)	0.838 (0.044)	0 (0)	0.162 (0.044)
0.25	1 (0)	0.185 (0.05)	0.815 (0.05)	0 (0)	0.185 (0.05)
0.3	1 (0)	0.187 (0.049)	0.813 (0.049)	0 (0)	0.187 (0.049)
0.35	1 (0)	0.211 (0.047)	0.789 (0.047)	0 (0)	0.21 (0.047)
0.4	0.999 (0.001)	0.257 (0.046)	0.743 (0.046)	0.001 (0.001)	0.255 (0.046)
0.45	0.997 (0.002)	0.341 (0.054)	0.659 (0.054)	0.003 (0.002)	0.338 (0.054)
0.5	0.997 (0.002)	0.356 (0.059)	0.644 (0.059)	0.003 (0.002)	0.352 (0.059)
0.55	0.996 (0.002)	0.36 (0.06)	0.64 (0.06)	0.004 (0.002)	0.356 (0.06)
0.6	0.994 (0.002)	0.367 (0.058)	0.633 (0.058)	0.006 (0.002)	0.361 (0.058)
0.65	0.992 (0.002)	0.376 (0.058)	0.624 (0.058)	0.008 (0.002)	0.368 (0.058)
0.7	0.988 (0.003)	0.393 (0.056)	0.607 (0.056)	0.012 (0.003)	0.38 (0.056)
0.75	0.977 (0.005)	0.414 (0.054)	0.586 (0.054)	0.023 (0.005)	0.391 (0.054)
0.8	0.956 (0.003)	0.468 (0.06)	0.532 (0.06)	0.044 (0.003)	0.424 (0.06)
0.85	0.914 (0.006)	0.55 (0.051)	0.45 (0.051)	0.086 (0.006)	0.464 (0.051)
0.9	0.827 (0.009)	0.685 (0.048)	0.315 (0.048)	0.173 (0.009)	0.512 (0.045)
0.95	0.625 (0.014)	0.862 (0.041)	0.138 (0.041)	0.375 (0.014)	0.487 (0.036)
1	0.113 (0.01)	0.991 (0.009)	0.009 (0.009)	0.887 (0.01)	0.103 (0.011)

Table S3.1 B - Dynamic uplift suitability model, based only on thermal and orographic uplift potentials (AUC±sd = 0.695±0.024).

Threshold	Sensitivity	Specificity	Commission Error	Omission Error	TSS
0	1 (0)	0 (0)	1 (0)	0 (0)	0 (0)
0.05	1 (0)	0 (0)	1 (0)	0 (0)	0 (0)
0.1	1 (0)	0 (0)	1 (0)	0 (0)	0 (0)
0.15	1 (0)	0.002 (0.004)	0.998 (0.004)	0 (0)	0.002 (0.004)
0.2	1 (0)	0.004 (0.004)	0.996 (0.004)	0 (0)	0.004 (0.004)
0.25	1 (0)	0.007 (0.005)	0.993 (0.005)	0 (0)	0.007 (0.005)
0.3	1 (0)	0.018 (0.012)	0.982 (0.012)	0 (0)	0.018 (0.012)
0.35	1 (0)	0.025 (0.016)	0.975 (0.016)	0 (0)	0.025 (0.016)
0.4	0.999 (0)	0.03 (0.016)	0.97 (0.016)	0.001 (0)	0.03 (0.016)
0.45	0.999 (0.001)	0.039 (0.013)	0.961 (0.013)	0.001 (0.001)	0.039 (0.013)
0.5	0.998 (0)	0.053 (0.008)	0.947 (0.008)	0.002 (0)	0.051 (0.008)
0.55	0.996 (0.001)	0.069 (0.015)	0.931 (0.015)	0.004 (0.001)	0.065 (0.014)
0.6	0.994 (0.001)	0.096 (0.016)	0.904 (0.016)	0.006 (0.001)	0.09 (0.017)
0.65	0.99 (0.002)	0.127 (0.017)	0.873 (0.017)	0.01 (0.002)	0.117 (0.018)
0.7	0.984 (0.003)	0.157 (0.026)	0.843 (0.026)	0.016 (0.003)	0.141 (0.026)
0.75	0.973 (0.005)	0.203 (0.033)	0.797 (0.033)	0.027 (0.005)	0.176 (0.033)
0.8	0.954 (0.006)	0.276 (0.039)	0.724 (0.039)	0.046 (0.006)	0.23 (0.04)
0.85	0.919 (0.009)	0.358 (0.047)	0.642 (0.047)	0.081 (0.009)	0.277 (0.049)
0.9	0.844 (0.008)	0.487 (0.045)	0.513 (0.045)	0.156 (0.008)	0.331 (0.045)
0.95	0.459 (0.018)	0.737 (0.033)	0.263 (0.033)	0.541 (0.018)	0.196 (0.039)
1	0.026 (0.005)	0.985 (0.014)	0.015 (0.014)	0.974 (0.005)	0.011 (0.015)

Table S3.1 C - Combined uplift suitability model, based on both static and dynamic predictors (AUC±sd = 0.862±0.016).

Threshold	Sensitivity	Specificity	Commission Error	Omission Error	TSS
0	1 (0)	0.001 (0.003)	0.999 (0.003)	0 (0)	0.001 (0.003)
0.05	1 (0)	0.051 (0.019)	0.949 (0.019)	0 (0)	0.051 (0.019)
0.1	1 (0)	0.075 (0.032)	0.925 (0.032)	0 (0)	0.075 (0.032)
0.15	1 (0)	0.107 (0.027)	0.893 (0.027)	0 (0)	0.106 (0.027)
0.2	1 (0)	0.133 (0.024)	0.867 (0.024)	0 (0)	0.133 (0.024)
0.25	1 (0)	0.157 (0.02)	0.843 (0.02)	0 (0)	0.157 (0.02)
0.3	1 (0)	0.169 (0.024)	0.831 (0.024)	0 (0)	0.169 (0.024)
0.35	1 (0)	0.192 (0.023)	0.808 (0.023)	0 (0)	0.191 (0.023)
0.4	0.999 (0)	0.234 (0.03)	0.766 (0.03)	0.001 (0)	0.234 (0.03)
0.45	0.999 (0.001)	0.287 (0.041)	0.713 (0.041)	0.001 (0.001)	0.285 (0.04)
0.5	0.998 (0.001)	0.319 (0.032)	0.681 (0.032)	0.002 (0.001)	0.317 (0.032)
0.55	0.998 (0.001)	0.341 (0.037)	0.659 (0.037)	0.002 (0.001)	0.339 (0.036)
0.6	0.997 (0.001)	0.356 (0.034)	0.644 (0.034)	0.003 (0.001)	0.352 (0.033)
0.65	0.995 (0.001)	0.375 (0.037)	0.625 (0.037)	0.005 (0.001)	0.37 (0.036)
0.7	0.992 (0.002)	0.392 (0.038)	0.608 (0.038)	0.008 (0.002)	0.384 (0.038)
0.75	0.983 (0.003)	0.417 (0.039)	0.583 (0.039)	0.017 (0.003)	0.4 (0.038)
0.8	0.962 (0.005)	0.471 (0.036)	0.529 (0.036)	0.038 (0.005)	0.433 (0.033)
0.85	0.92 (0.008)	0.557 (0.038)	0.443 (0.038)	0.08 (0.008)	0.477 (0.035)
0.9	0.829 (0.014)	0.706 (0.036)	0.294 (0.036)	0.171 (0.014)	0.535 (0.036)
0.95	0.609 (0.014)	0.878 (0.032)	0.122 (0.032)	0.391 (0.014)	0.488 (0.022)
1	0.055 (0.004)	1 (0)	0 (0)	0.945 (0.004)	0.055 (0.004)

Supporting figures for the models' output

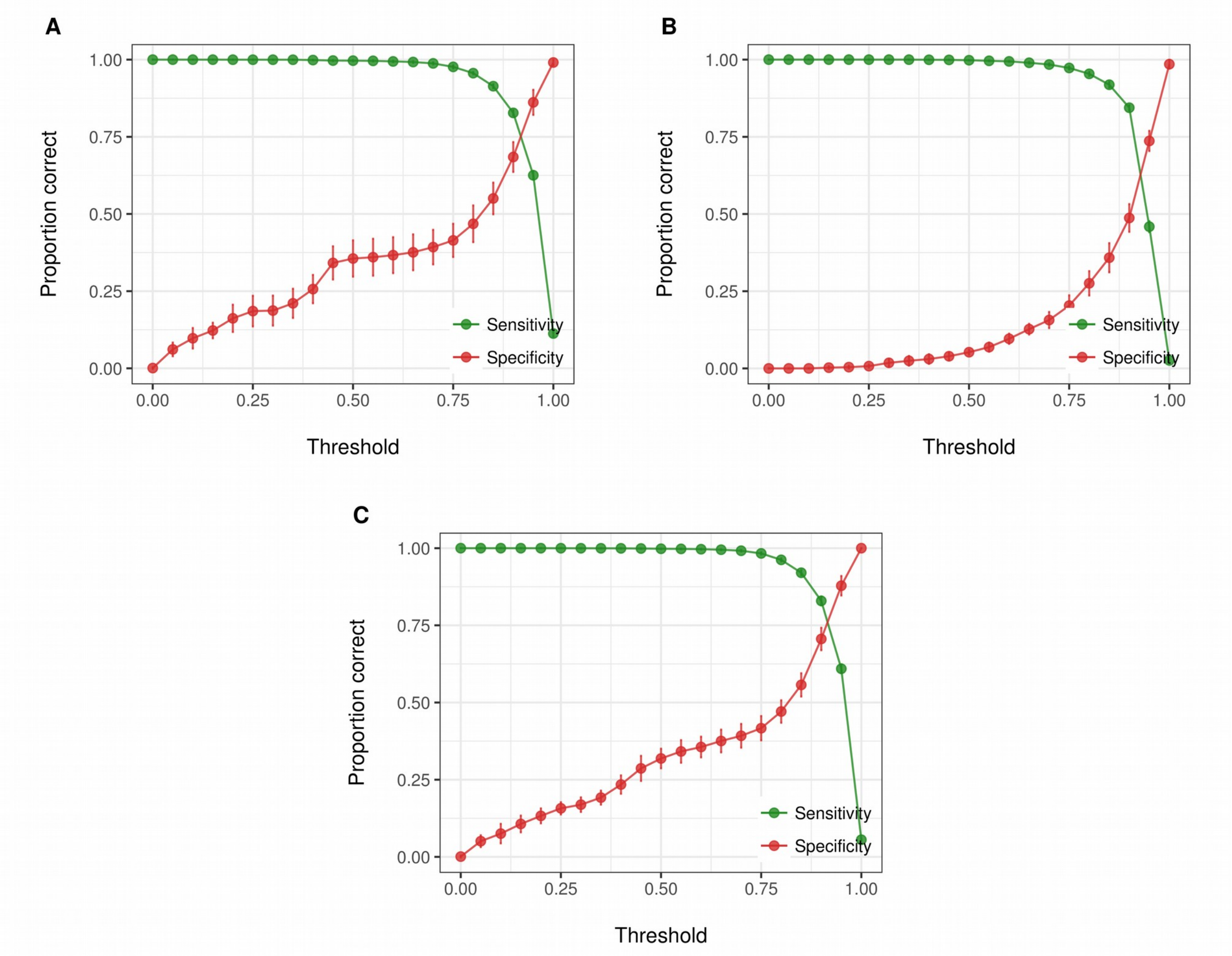

Figure S3.2 - Accuracy of the three uplift suitability models, static (A), dynamic (B) and combined (C), in terms of sensitivity (proportion of soaring locations correctly classified, in green) and specificity (proportion of flapping locations correctly classified, in red) at different thresholds values. The solid points represent the value of Sensitivity and Specificity, averaged across the ten runs of each model, at a threshold that maximize the value of the True Skill Statistics.

A - Static model

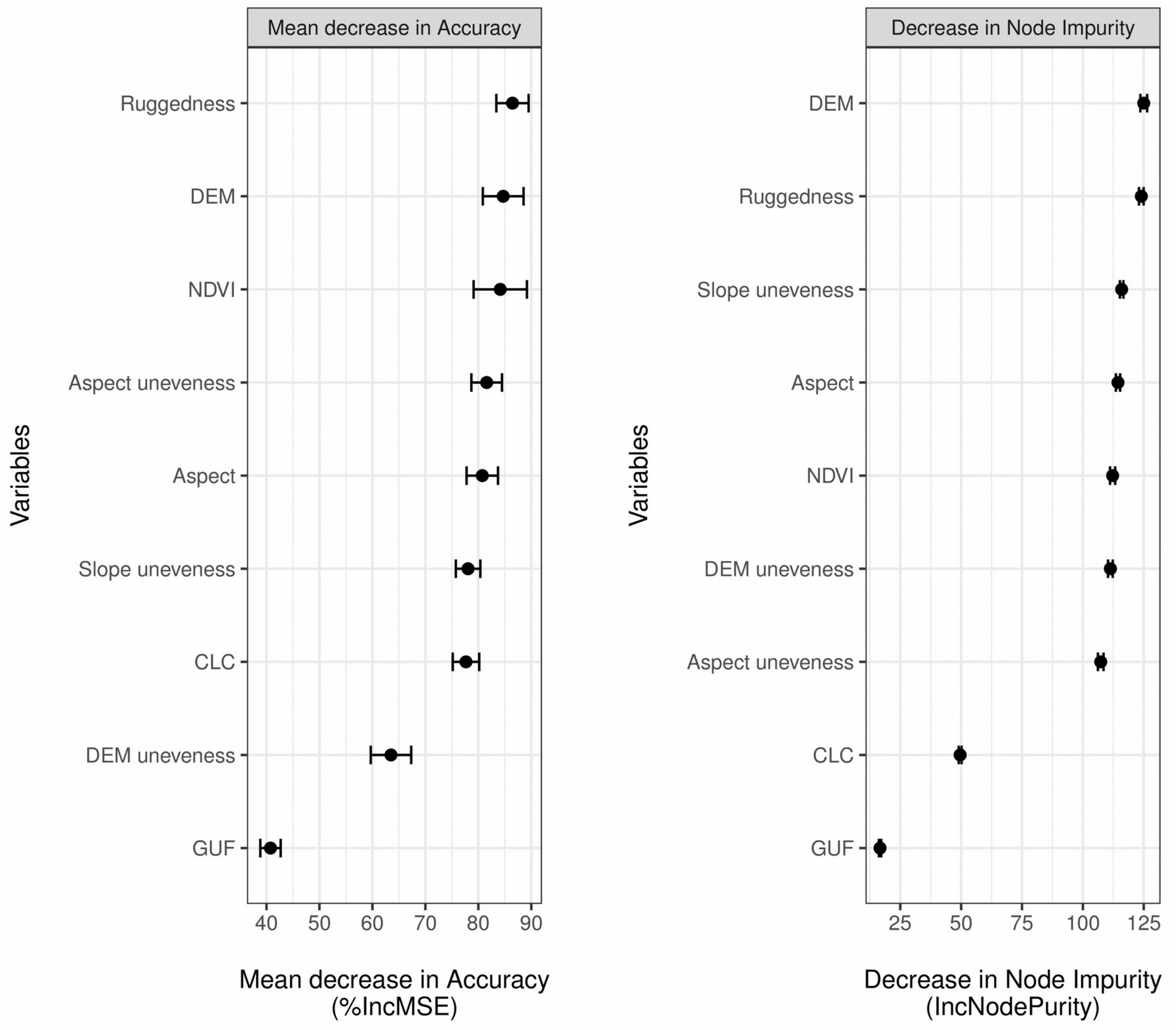

B - Dynamic model

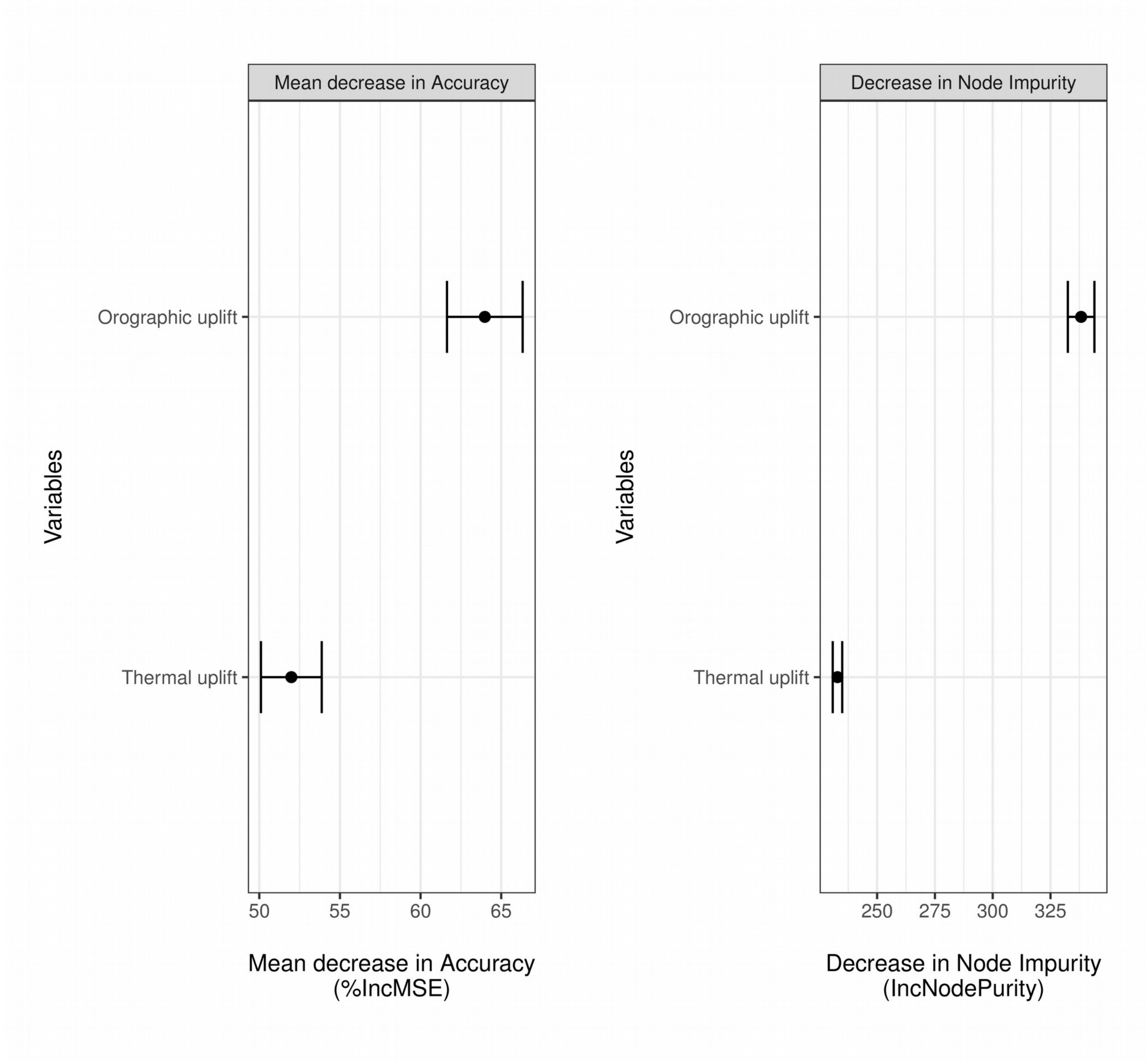

C - Combined model

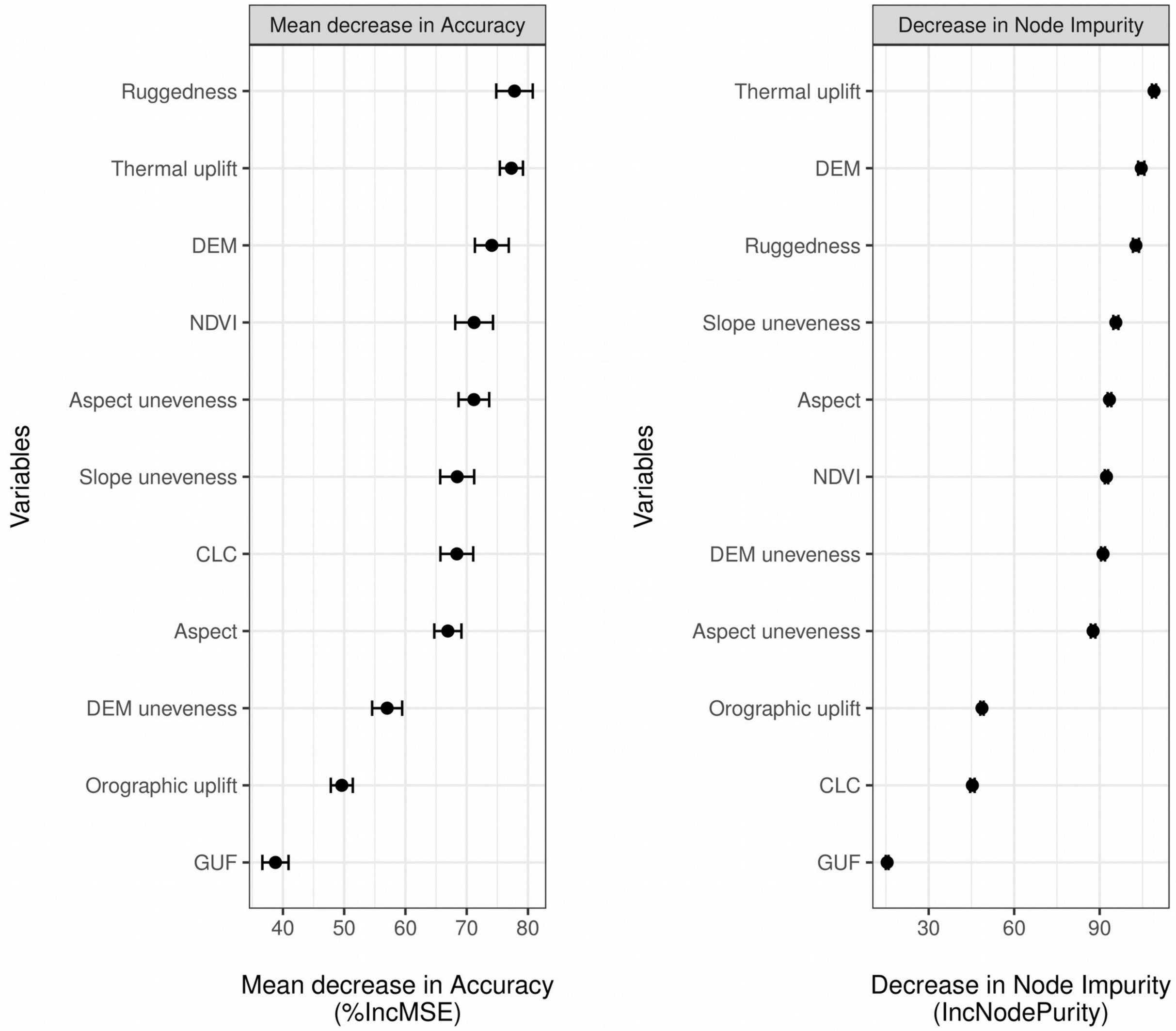

Figure S3.3 - Variable importance. Contributions of the different variables to the static (A), dynamic (B) and combined (C) uplift suitability models, measured in terms of mean decrease in accuracy (left) and decrease in node impurity (right).

S4 – Uplift intensity model

Table S4.1 - Summary of the three uplift intensity models (generalized additive models).

	Response: sqrt(Vertical Speed)		
	Static Uplift intensity model	*Dynamic Uplift intensity model*	*Combined Uplift intensity model*
**Parametric coefficients:** Estimate (C.I.)			
Intercept	0.898 (0.866, 0.929)	0.765 (0.759, 0.771)	0.719 (0.688, 0.751)
Aspect unevenness	0.0001 (0.00000, 0.0001)		0.0001 (0.00000, 0.0001)
DEM unevenness	0.001 (-0.0002, 0.002)		0.001 (-0.0004, 0.002)
Slope unevenness	0.005 (0.002, 0.008)		0.005 (0.002, 0.008)
GUF	0.013 (-0.001, 0.028)		0.022 (0.008, 0.037)
CLC 1 - dumps	-0.155 (-0.310, -0.0004)		-0.039 (-0.190, 0.112)
CLC 2 - art. veg. areas	0.063 (0.023, 0.103)		0.055 (0.015, 0.094)
CLC 3 - arable lands	-0.018 (-0.049, 0.014)		-0.019 (-0.050, 0.012)
CLC 4 - perm. crops	-0.023 (-0.055, 0.009)		-0.016 (-0.047, 0.016)
CLC 5 - pastures	-0.034 (-0.068, 0.0001)		-0.028 (-0.062, 0.005)
CLC 6 - het. agr. areas	-0.017 (-0.049, 0.015)		-0.012 (-0.044, 0.019)
CLC 7 - forest	0.014 (-0.018, 0.046)		0.009 (-0.022, 0.041)
CLC 8 - Shrubs	0.024 (-0.008, 0.057)		0.022 (-0.011, 0.054)
CLC 10 - wet. marshes	-0.050 (-0.088, -0.012)		-0.026 (-0.064, 0.012)
CLC 11 - marine wet. salines	0.002 (-0.032, 0.036)		0.007 (-0.027, 0.040)
CLC 12 - rivers	-0.020 (-0.070, 0.029)		-0.026 (-0.075, 0.023)
CLC 13 - wat. bodies lakes	-0.102 (-0.152, -0.051)		-0.093 (-0.143, -0.044)
CLC 14 - coast. lagoons est.	0.060 (0.014, 0.105)		0.069 (0.024, 0.114)
CLC 15 - sea	0.006 (-0.450, 0.462)		-0.004 (-0.449, 0.441)
CLC 16 - urban areas	-0.013 (-0.046, 0.020)		-0.017 (-0.049, 0.016)
Therm. uplift pot.		0.124 (0.119, 0.129)	0.161 (0.156, 0.166)
Orog. uplift pot.		0.042 (0.029, 0.056)	0.063 (0.049, 0.077)
**Smooth terms:** F (Effective df)			
s(Aspect)	4.057 (3.040)		2.080 (2.545)
s(DEM)	49.019 (6.978)		40.020 (6.901)
s(Ruggedness)	1.863 (4.443)		4.510 (4.875)
s(NDVI)	18.068 (6.710)		21.650 (4.277)
s(Latitude)	60.916 (8.881)		91.420 (8.885)
Observations	76383	78307	74946
Adjusted R2	0.032	0.032	0.083
AIC (df)	46575.00 (54.745)	49636.88 (4)	42003.95 (53.19181)

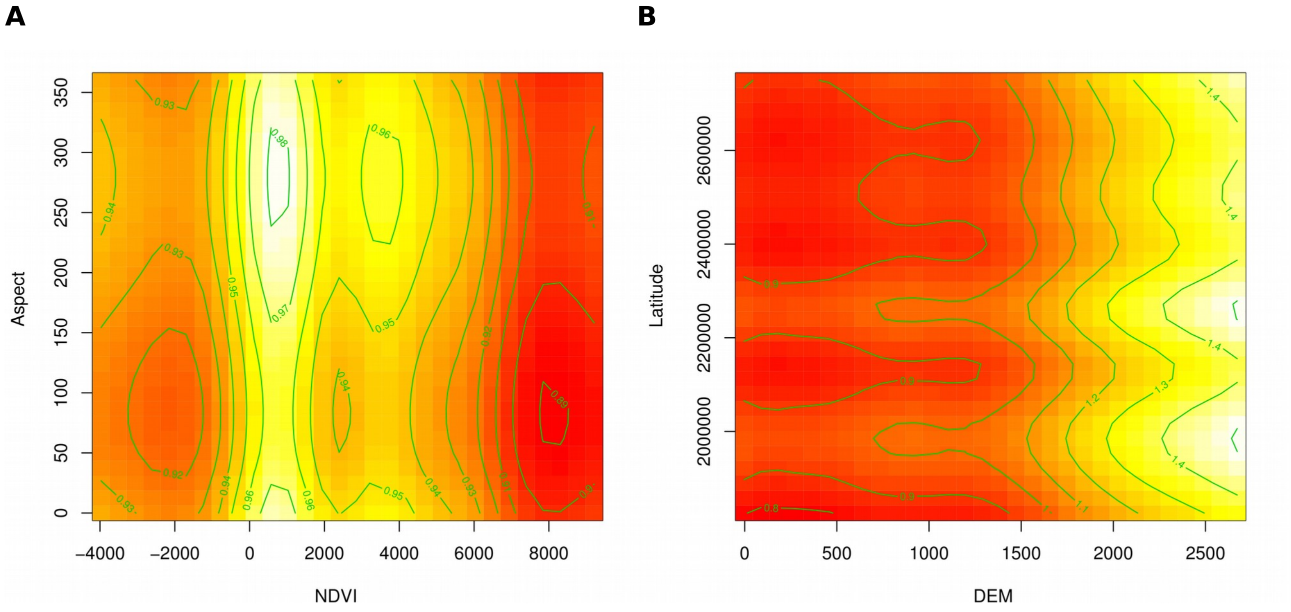

Figure S4.1 - Non linear relationship between some of the environmental predictors included as smooth terms in the static uplift intensity model (generalized additive model) and the response variable (vertical speed). In (A) the effect of NDVI and aspect, whereas in (B) the effect of DEM and latitude on the vertical speed. In lighter colours, combination of variables' values that predict a higher vertical speed.

S5 – Static energy landscape

Table 5.1 - Output of the Linear Mixed Model analysing the mean daily ODBA as a function of the mean daily uplift suitability (as predicted by the static uplift suitability model).

	**Response variable: sqrt(Mean Daily ODBA)**
**Fixed effects:** Estimate (C.I.)	
Intercept	3.548 (0.166)
Mean daily uplift suitability	-2.252 (0.189)
**Random effects:** Variance (St. Dev., Corr.)	
Intercept	0.990 (0.995)
Mean daily uplift suitability	1.248 (1.117, -1.00)
Residual	0.055 (0.234)
Observations	823
Groups (Individuals)	59
AIC	46.233
AIC (null model)	114.254
ΔAIC = AIC – AICnull	-68.021
Marginal R^2	0.306
Conditional R^2	0.426

REFERENCES – SUPPLEMENTARY MATERIAL

1. EEA. 2013 European Environment Agency - Digital Elevation Model over Europe (EU-DEM). *Accessed March 2017*. See https://www.eea.europa.eu/data-and-maps/data/eu-dem.
2. Hijmans RJ. 2016 *raster: Geographic Data Analysis and Modeling*. R package version 2.5-8. See https://cran.r-project.org/package=raster.
3. Horn BKP. 1981 Hill shading and the reflectance map. *Proc. IEEE* **69**, 14–47.
4. Masek JG, Vermote EF, Saleous N, Wolfe R, Hall FG, Huemmrich F, Gao F, Kutler J, Lim T. 2006 A Landsat surface reflectance data set for North America, 1990-100. *IEEE Geosci. Remote Sens. Lett.* **3**, 68–72. (doi:10.1109/LGRS.2005.857030)
5. EEA. 2012 European Environment Agency - Corine Land Cover 2012 raster data. *Accessed March 2017*. See https://www.eea.europa.eu/data-and-maps/data/clc-2012-raster.
6. DLR. 2011 Earth Observation Center – Global Urban Footprint. *Accessed March 2017*. See http://www.dlr.de/eoc/en/desktopdefault.aspx/tabid-11725/20508_read-47944/.
7. Dodge S *et al.* 2013 The environmental-data automated track annotation (Env-DATA) system: linking animal tracks with environmental data. *Mov. Ecol.* **1**, 3. (doi:10.1186/2051-3933-1-3)
8. Bohrer G, Brandes D, Mandel JT, Bildstein KL, Miller TA, Lanzone M, Katzner T, Maisonneuve C, Tremblay JA. 2012 Estimating updraft velocity components over large spatial scales: Contrasting migration strategies of golden eagles and turkey vultures. *Ecol. Lett.* **15**, 96–103. (doi:10.1111/j.1461-0248.2011.01713.x)
9. U.S. Geological Survey. In press. USGS Earth Resources Observation and Science (EROS) Center Science Processing Architecture (ESPA) On Demand Interface. *Accessed March 2017*. See https://espa.cr.usgs.gov/.
10. Barbet-Massin M, Jiguet F, Albert CH, Thuiller W. 2012 Selecting pseudo-absences for species distribution models: how, where and how many? *Methods Ecol. Evol.* **3**, 327–338. (doi:10.1111/j.2041-210X.2011.00172.x)

11. Franklin J. 2009 *Mapping species distributions. Spatial inference and prediction*. Cambridge University Press.
12. Allouche O, Tsoar A, Kadmon R. 2006 Assessing the accuracy of species distribution models: Prevalence, kappa and the true skill statistic (TSS). *J. Appl. Ecol.* **43**, 1223–1232. (doi:10.1111/j.1365-2664.2006.01214.x)
13. Franklin J, Wejnert KE, Hathaway SA, Rochester CJ, Fisher RN. 2009 Effect of species rarity on the accuracy of species distribution models for reptiles and amphibians in southern California. *Divers. Distrib.* **15**, 167–177. (doi:10.1111/j.1472-4642.2008.00536.x)
